# Disparities in Disease-Specific Remaining Life Expectancy Among Medicare Beneficiaries in the United States

**DOI:** 10.1093/geroni/igab046.1067

**Published:** 2021-12-17

**Authors:** Julia Kravchenko, Bin Yu

**Affiliations:** 1 Duke University, Durham, North Carolina, United States; 2 Duke University, Duke University/Durham, North Carolina, United States

## Abstract

Racial and geographic disparities in life expectancy (LE) in the US are a persistent problem. We used 5% Medicare Claims for 2000-2017 to investigate the patterns of remaining LE (RLE) in the U.S. with the highest and the lowest LE. RLEs in race-/ethnicity specific populations aged 65+ were calculated in patients with specific diseases and in the total population using the area under the Kaplan-Meier estimator. The Cox model was used to investigate the effect of state-specific residence on total LE and RLE. Between-the-states differences in RLE were most pronounced for cerebrovascular disease, atherosclerotic heart disease, breast and prostate cancer. RLE was the lowest for lung cancer and sepsis, followed by Alzheimer’s disease, dementia, pneumonia, and heart failure. RLE for myocardial infarction and cerebrovascular disease decreased over time, while for renal failure, diabetes, atherosclerotic heart disease, and cancers of breast and prostate RLE increased.

